# C1 lateral mass reconstruction using a titanium cage following total resection of the aneurysmal bone cyst: illustrative cases and literature insights

**DOI:** 10.1097/MS9.0000000000002785

**Published:** 2024-12-12

**Authors:** Kaveh Ebrahimzadeh, Mohammad Mirahmadi Eraghi, Mohammad Hallajnejad, Mohammad Ansari, Hesameddin Hosseini Tavasol, Seyed Ali Mousavinejad, Mohammad Samadian

**Affiliations:** a Skull Base Research Center, Loghman Hakim Hospital, Shahid Beheshti University of Medical Sciences, Tehran, Iran; b Brain and Spinal Cord Injury Research Center, Neuroscience Institute, Tehran University of Medical Sciences, Tehran, Iran

**Keywords:** Aneurysmal bone cyst, Atlas, craniovertebral junction, lateral mass reconstruction, titanium mesh cage

## Abstract

**Introduction:**

There are limited reports of C1 lateral mass reconstruction using a titanium mesh cage following the surgical removal of an aneurysmal bone cyst (ABC). We describe two uncommon and challenging cases of C1 ABC, highlighting the obstacles and complexities involved in selecting the appropriate approach for tumor resection, C1 stabilization, and reconstruction.

**Case discussion:**

***Case 1**:* A 12-year-old boy presented with 3 months of progressive upper cervical and occipital pain with no history of trauma. A heterogeneous lytic lesion with fluid-fluid levels in the right lateral mass of the atlas was detected. A gross total resection(GTR) of the C1 lateral mass ABC was performed through a posterior approach, followed by constructing the C1 lateral mass. ***Case 2***: An 11-year-old girl presented with cervical pain for the past 6 months. A cervical computed tomography (CT) scan without contrast revealed a lytic-expansile mass lesion with bony erosion in the right transverse process of the C1 vertebra. A similar surgical plan was tailored for this patient. A follow-up examination at 6 weeks demonstrated complete pain relief, and routine neurologic evaluations were uneventful.

**Discussion:**

ABCs are uncommon, non-malignant, and highly vascular tumors, accounting for approximately 1% of all bone tumors and 15% of primary spinal tumors. The treatment of choice in this region is total resection, followed by C1 reconstruction.

**Conclusion:**

C1 lateral mass reconstruction using an expandable cage with vertebral artery (VA) preservation is recommended for extensive C1 lateral mass resection due to ABC.

## Introduction

Aneurysmal bone cysts (ABCs) are uncommon, non-malignant, and highly vascular tumors, constituting about 1% of all bone tumors, and 15% of primary tumors arise from the spinal cord^[1-3]^. ABCs often involve posterior elements of the spine, with a higher prevalence in the lumbar spine (45%), followed by the cervical and thoracic regions^[4,5]^. ABCs arise from the cervical spine and comprise about one-third of spinal ABC cases, while C1 involvement was reported in only 1% of instances^[6]^. ABCs can result in pain, deformity, and instability, necessitating appropriate intervention, and predominantly affect the pediatric population^[1,2,5]^. This study presents the third report of atlas lateral mass reconstruction using a titanium mesh cage following the surgical resection of ABC, elucidating the challenges associated with selecting the proper approach for tumor resection, C1 stabilization, and reconstruction.

**Highlights**

- Total resection of a C1 aneurysmal bone cyst and lateral mass reconstruction using a titanium mesh cage provides a viable approach for managing this rare condition.
- Preoperative digital subtraction angiography and balloon occlusion tests facilitate the effective surgical removal of C1 ABCs.
- Titanium mesh cage reconstruction provides biomechanical stability, restoring load-bearing properties after C1 lateral mass resection.

## Illustrative case

### History and examination

#### Case 1

A 12-year-old boy presented to our center with 3 months of progressive upper cervical and occipital pain with no history of trauma. On examination, his head was in the cock-robin position with significant restriction of the cervical range of motion in all directions. There was a palpable non-tender mass in the right superior posterolateral of the cervical region. The neurological examination was found to be uneventful, with typical motor and sensory evaluations.

#### Case 2

An 11-year-old girl has been suffering from cervical pain for the past 6 months. She experienced localized suboccipital and upper cervical pain without radiculopathy to the upper limbs, with progression during the month prior to admission. She had no history of medical disorders, and her family history was also unremarkable. She had not received any medication before. The physical examination was normal, including cranial nerves, muscle forces, and sensory analysis. This case report has been reported in line with SCARE guidelines^[7]^.

### Diagnostic evaluation

#### Case 1

**Figure 1.**
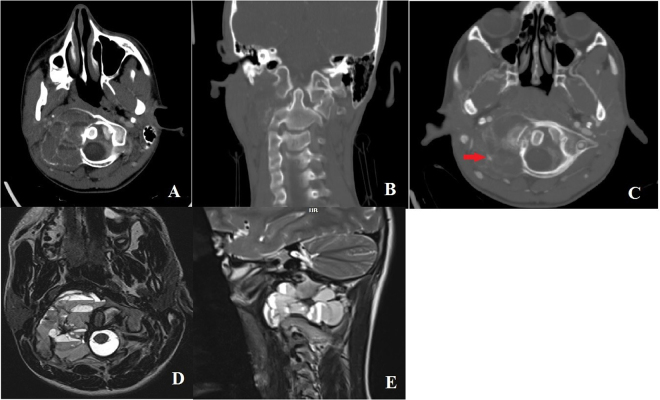
(A) an axial CT scan showing an expansile lesion with septations in an egg-shell appearance involving the atlas lateral mass, with anterior and posterior arc destruction and retropharyngeal extension. (B) a coronal CT revealed destruction of the right side lateral mass and C1-C2 dislocation on the left side. (C) the right VA is encased by the lesion based on CT-angiography of VAs (red arrow). (D) axial T2-weighted MRI demonstrates a cystic lesion with a fluid-fluid appearance without evidence of cord compression. (E) the sagittal view depicts the anterior-posterior extension of the tumor to the retropharyngeal space.

The patient’s computed tomography (CT) scan of the craniocervical junction region revealed a 5 × 5 × 4 cm expansile lytic lesion embedded in the right lateral mass of the C1, extending into both the anterior and posterior arches. It also revealed rightward rotational and anterior subluxation of the atlas relative to the axis, accompanied by some degree of odontoid basilar invagination (Fig. 1A and B). Cervical CT angiography showed that the tumor encased the V3 segment of the right vertebral artery (VA) (Fig. 1C). Magnetic resonance imaging (MRI) demonstrated a heterogeneous mass with fluid-fluid levels on both T1 and T2-weighted sequences (Fig. 1D and E).

Preoperative cerebral angiography demonstrated co-dominant VAs with significant narrowing of the right VA at the V3 segment and tumor encasement. There was mild tumor blush with no prominent feeding arteries, so embolization was not undertaken. A balloon occlusion test (BOT) was performed, and there was no neurologic deficit after 30 minutes of occlusion of the right VA. Radiographic findings suggested ABC, eosinophilic granuloma, and giant cell tumor.

#### Case 2

A defined lytic-expansile mass lesion with bony erosion measuring 49 × 36 × 30 mm in the right-sided transverse process of C1 was noted in the cervical CT scan without contrast. MRI evaluation with and without contrast injection revealed a well-defined enhancing mass lesion in the right lateral mass of C1 with encasement of the right VA measuring about 49 × 36 mm. The mass had close proximity to the thecal sac without intrathecal extension (Figs. 3, 4). The core needle biopsy evaluation revealed a giant cell-rich bone-forming neoplasm, while the post-operative pathology report showed a giant cell-rich tumor compatible with solid ABC.

### Operative technique

Several therapeutic options, including percutaneous CT-guided biopsy, open tumor resection, and craniocervical stabilization, were considered. Due to relentless pain and the degree of atlas destruction and craniocervical instability, the patients and their parents were recommended for resection of the lesion and stabilization.

Following fiber optic intubation and general anesthesia, the patient was positioned prone in a Mayfield head holder. Intraoperative neuromonitoring was performed throughout the operation with somatosensory evoked and motor evoked potential. A hockey stick incision was made starting at the C4 spinous process, extending through the midline above the inion, directed laterally 4 centimeters, and inferiorly along the mastoid process, ending 1 centimeter below the mastoid tip. The dorsal fascia was exposed in the midline, and muscles were carefully dissected from the bone using a subperiosteal fashion to expose occipital bone, C1 posterior arch, and C2-C4 spinous process and lamina. The tumor, which had extensively replaced the right side of the posterior arch and lateral mass of C1, was visualized following subperiosteal dissection. As preoperative imaging demonstrated the encasement of the VA, and since the BOT was tolerable by the patient, and in order to reach gross total resection (GTR), we preferred two-point ligation of the Right VA to achieve GTR. We exposed the proximal part of the V3 segment at the exit point of C2 transverse foramina and ligated the right VA using hemoclips.

The second clip was placed at the VA proximal to the dural entrance. The right C1 and C2 roots were identified and ligated for further exposure. The tumor was dissected without entering the tumor mass. Resection continued anteriorly toward the retropharyngeal space end; en bloc resection was eventually achieved. Following the resection, the superior articular surface of C2 and the inferior aspect of the occipital condyle were distinguishable without evidence of erosion and destruction.

**Figure 2.**
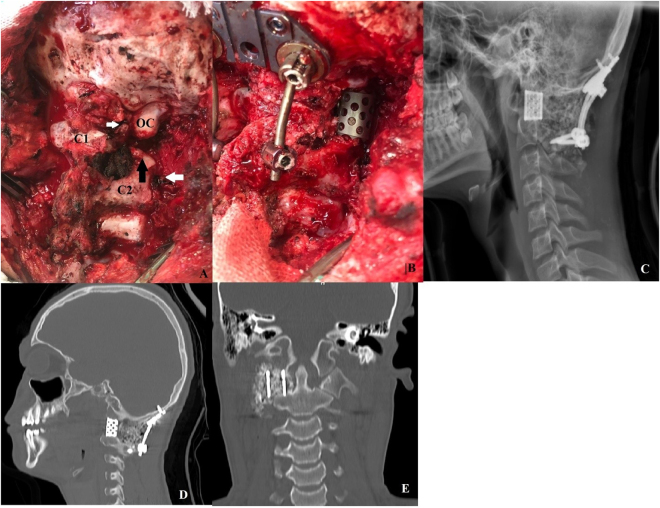
A: the intraoperative view shows a cavity due to total resection of the tumor (OC: occipital condyle, C1 the remaining of the atlas posterior arc after tumor resection, C2, and black arrow: the superior articular surface of the axis, white arrows: proximal and distal part of the VA that was ligated). B: after drilling and occipitocervial fixation, an appropriately sized mesh cage was fixed between the axis articular surface and the occipital condyle. C: postoperative lateral plain X-ray showing evidence of reconstruction, D and E: sagittal, and coronal view on CT scan demonstrating the mesh cage between the axis articular surface and the occipital condyle.

At this point, craniocervical junction stabilization and C1 lateral mass reconstruction were initiated. A midline occipital plate was placed flush against the occipital bone with a side screw based on a measurement from the preoperative CT scan. Using the technique described by Neil^[8]^ for inserting bilateral C2 laminar screws, we inserted polyaxial screws that were 4.5 mm in diameter and 32 mm in length. Posterior reconstruction finalized using rod placement. Then, the right articular surface of C2 and the occipital condyle were decorticated with the high-speed drill. An appropriately sized titanium mesh cage was placed between the superior articular surface of C2 and the inferior aspect of the occipital condyle to reestablish the load-bearing feature of the C1 lateral mass. For further stabilization, Pedicle screws were inserted in the left side of the C1 and right side of C2, and C3 and C4 vertebrae were instrumented with lateral mass bilaterally for the second case (Figs. 2, 5, 6, 7).

The bony surface was decorticated, and allograft bone was placed. Since the right VA had already been ligated, no pedicle or lateral mass screw was fixed on the left side to avoid potential injury to the patient’s left-sided VA. At the end of the surgery, the total amount of bleeding was estimated to be about 600.

#### Case 2

**Figure 3.**
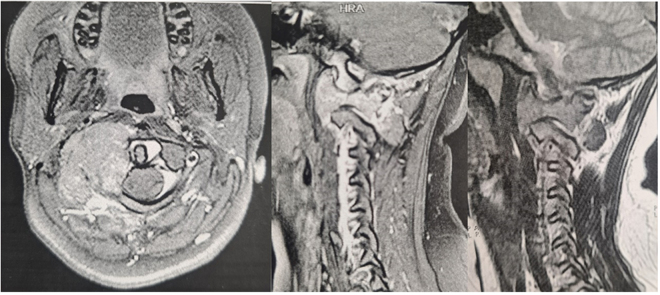
Cervical spine MRI demonstrating a well-defined mass measured 49 × 36 mm in right lateral mass of atlas with encasement of right VA enhancing after gadolinium injection. Left side: lateral view without gadolinium injection; middle: lateral view following gadolinium injection; right: axial view following gadolinium injection.

**Figure 4.**
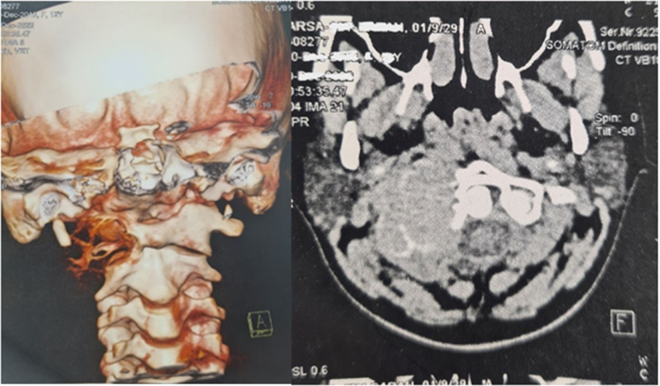
The cervical CT scan without contrast shows a 30 × 36 × 49 mm lytic-expansile mass lesion with bony erosion in the right transverse process of Atlas. Left side: axial view; right: 3D reconstruction.

**Figure 5.**
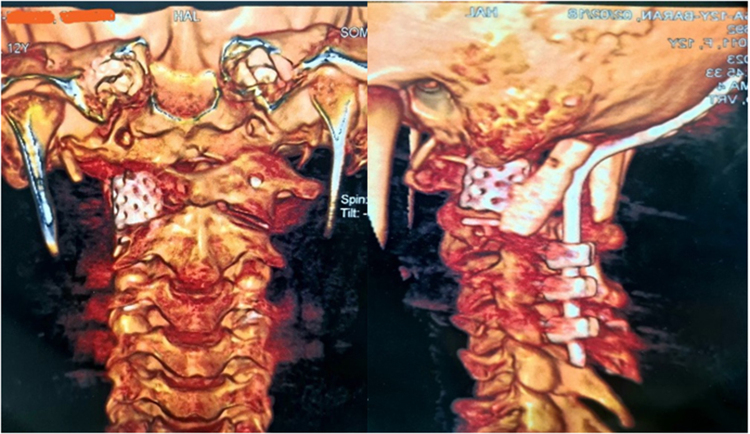
Cervical CT scan without contrast injection with 3D reconstruction showing resection of the right C1 lateral mass ABC and reconstruction using titanium mesh cage. Left: lateral view; right: anterior view.

**Figure 6.**
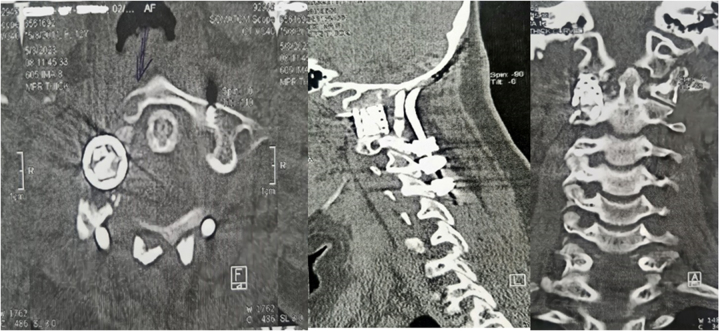
Cervical CT scan without contrast injection showing resection of the right C1 lateral mass ABC and reconstruction with titanium mesh cage. Right: anterior view; middle: lateral view; left: axial view.

**Figure 7.**
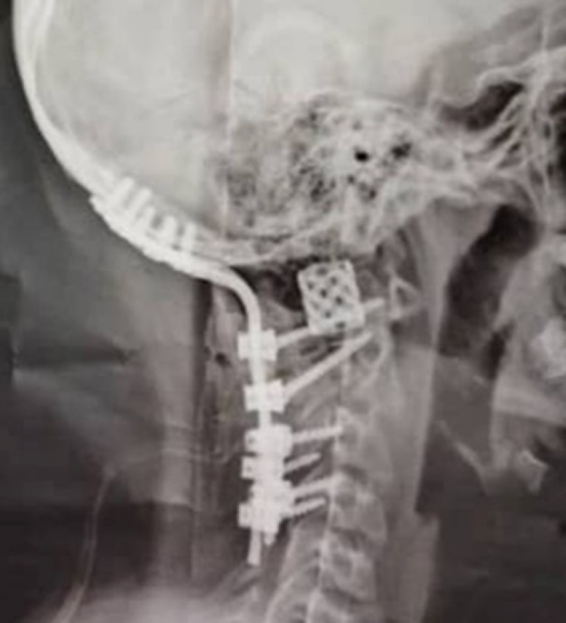
The cervical X-ray (lateral view) shows resection of the right C1 lateral mass ABC and reconstruction with a titanium mesh cage. Pedicle screws were inserted in the left side of the C1 and right side of C2, and the C3,4 vertebra was instrumented with lateral mass bilaterally. Finally, the patient underwent occipitocervical fusion from the occipital bone to the C4 vertebrae to reach craniocervical stability.

A percutaneous biopsy on preoperative week 3 confirmed the diagnosis of ABC. We followed the same anesthesia protocol, and the patient was placed in prone position. A midline incision was made from the inion to the C5, and the patient’s fascia and muscles were meticulously dissected. The tumor had encased the right-sided VA in our preoperative evaluations. A minor bleeding occurred during the microsurgical tumor removal, which was effectively controlled by cautery. After achieving GTR and hemostasis, a micro drill was used to decorticate the lower aspect of the occipital condyle and the upper lateral aspect of C2 on the right side. An appropriately sized mesh cage with allograft bone was inserted. A lateral screw on the left side of C1, bilateral pedicle screws on C2, and bilateral lateral screws on C3 and C4, using a rod plate, provided satisfactory stabilization. Through compression maneuvers, the cage was then secured firmly. A fibular allograft was inserted bilaterally between the occipital bone and the C2 lamina to achieve the fusion. Finally, the total estimated blood loss was approximately 650 cc. (see Figs. 3–7).

### Postoperative course and outcome

#### Case 1

The patient was discharged on postoperative day 4 without any medical or neurological complications. A cranial cervical-thoracic brace was prescribed for 3 months. Complete pain relief with routine neurologic evaluations was achieved at postoperative week 6. Follow-up radiographs revealed the excellent position of implants (Fig. 2C–E). Post-op imaging revealed C1 lateral mass reconstruction using a mesh cage and also occipital plate and bilateral C2 laminar screws fixed via rod device.

#### Case 2

The patient was discharged a few days post-operatively with a cervicothoracic immobilizing brace without any complications during the hospital stay. She was followed for approximately 6 months, during which her cervical pain was alleviated. The CT scan without contrast demonstrated that the C1 lateral mass was resected and replaced by a titanium mesh cage. Pedicle screws were inserted in the left side of the C1 and right side of C2, and C3 and C4 vertebrae were instrumented with lateral mass bilaterally. Finally, the patient underwent occipitocervical fusion from the occipital bone to the C4 vertebra to correct the singular/plural inconsistency to reach craniocervical stability (Figs. 5, 6, 7).

## Discussion

Van Arsdale is known for the initial description of ABC in 1893, whereas termaneurysmal bone cyst was introduced by Jaffe and Lichtenstein in 1942^[9]^. ABCs represent uncommon benign tumors, with an estimated prevalence of 0.14 cases per 100 000 individuals. These tumors predominantly affect the adolescent population, with a significant female predominance observed in the cervical spine (male-to-female ratio of 1:2)^[1,10]^. These lesions constitute 1.4% of all primary bone tumors, with long bones accounting for almost 10%–30% of spinal involvement^[11,12]^. Cervical involvement was reported in one-third of spinal reports; however, C1 involvement was presented in approximately 1% of cases^[6,13]^.

ABCs are characterized by blood-filled cystic cavities divided by connective tissue septa, which contain osteoclast-like giant cells, fibroblasts, and reactive bone^[14]^. These lesions can range from rapidly expanding and destructive to slow-growing, eventually resolving independently^[15,16]^.

The localized pain represents the most common initial presentation of ABCs^[1,2,5,17]^. ABCs may manifest with swelling, tenderness upon palpation, and deformity^[2,18]^. ABCs can cause severe local bone damage by exerting pressure on nearby structures, including the adjacent cord, nerve roots, VA, and soft tissues. Neurological deficits are found in 60%–70% of cases, ranging from mild radiculopathies and myelopathy to more pronounced ones^[5]^.

The diagnostic value of imaging features is noteworthy. Plain X-rays and CT scans may reveal expansive, well-defined lytic lesions with periosteal calcification resembling an “eggshell” and cortical thinning, often characterized as a “soap bubble” appearance^[19,20]^. Typical MRI findings indicate a multiseptated cystic lesion with fluid-fluid levels, appearing hypointense on T1-weighted and hyperintense on T2-weighted sequences, with contrast-enhancing septal walls^[19,20]^. The fluid-fluid levels place the ABC in the main differential diagnosis^[21]^. Other differential diagnoses may include hemangiomas, giant cell tumors, fibrous dysplasia, and metastatic deposits^[19]^.

Selective angiography enables the identification of feeder vessels. It also possesses therapeutic value through simultaneous lesion embolization, which reduces vascularity and minimizes intraoperative bleeding^[2,5]^.

ABCs are conventionally managed through surgical resection, with en bloc excision associated with less blood loss and lower recurrence rates^[6]^. Intralesional curettage also provides a choice; however, it is believed to be associated with a higher recurrence rate^[22]^. Zenonos et al.^[4]^ proposed that selective arterial embolization may be as effective as intralesional surgery, offering a less invasive and cost-effective option for small lesions. Complete resection provides the highest cure rate and thus remains the preferred treatment, especially when pathological fractures and neurological deficits are noted^[11,14,23]^. Surgery is preferred when spinal stability is compromised, and neurological signs are present^[1,24,25]^. Extensive tumor growth leading to deformity and instability may necessitate surgical resection accompanied by reconstruction using bone grafting and instrumented stabilization^[1,5,24,26]^.

Radiotherapy is known for its sclerosing effects on ABCs. However, it poses a higher risk of subsequent deformity, especially when combined with surgical curettage. While radiation alone can be effective, Capanna et al.^[15]^ reported increased recurrences when used in isolation, especially in pediatrics, with a potential risk of late malignancy in irradiated areas^[11,23]^.

C1 ABCs are extremely rare, constituting about 1% of all spinal ABCs^[6,27]^, and they are typically located in the lateral mass. Only two previous studies have described C1 lateral mass reconstruction using a titanium mesh cage after surgical removal of ABC (Table 1).

**Table 1:** Previous reports of C1 lateral mass reconstruction using the titanium cage following surgical resection of ABC

	Author/year	Age/gender	Presentation	Comorbidity	Region of C1 involvement	Radiologic findings	Surgical management	VA preservation	Follow-up
1	Wang (2009) (6)	12/F	Neck pain	None	Right lateral mass and anterior arch	The CT scan demonstrated a lytic lesion (4.4 × 3 cm) in the right C1 lateral mass.	Lateral mass screws were bilaterally inserted at C3/4 using the Magrel technique.	The right VA was endovascularly occluded from the intradural portion down to the C2 segment.	At postoperative month 6, signs of fusion were evident, with bony trabeculae extending from the occipital condyle through the titanium.
							The Kerrison rongeurs were employed to remove the C1 posterior arch.		
									In the last follow-up, she had a neck visual analog scale score of 2 and a short form-36 physical component score of 42.
							The right VA, coiled, was mobilized, ligated using hemoclips, and transected through the coils. Ligations were performed on the right C1/C2 nerve roots.		
							The right lateral mass of the atlas was removed from the occipital condyle, extending through the superior articular pillar of C2 using a pituitary rongeur and curette.		
							Hydrogen peroxide provided hemostasis and local tumor control.		
							A 16-mm pyramesh cage, contoured to conform to the superior surface of the articular pillar of C2 lateral mass and the occipital condyle, was packed with autograft harvested from the iliac crest.		
							The C2 lateral mass and the occipital condyle were decorticated.		
							The screw was fixed through the cage to secure it to the patient’s occipital condyle.		
							Two 3.5-mm rods were positioned over the occipital plate and the lateral mass screws.		
						It was isodensity to bone on T1 and hyperdensity on T2 with heterogeneous enhancement.	The rib graft was divided into 2 halves; one right side situated from the occipital condyle to the C3 lateral mass and stabilized with microfracture screws. The other was inserted on the left and secured with a C2 sublaminar wire.		
							A cross-link provided additional stability.		
						Preoperative angiography demonstrated collateral blood flow from the tumor's feeding vessels to the anterior spinal artery.			
2	Neva(2017) (48)	18/F	Chronic headaches and progressive neck pain	Obesity and mitral valve repair	C1 posterior arch and lateral mass	The MRI revealed a cystic lesion 5.3 cm in size, affecting the posterior arch and the lateral mass on the right side.	A midline posterior approach complemented the intraoperative navigation	Preserved.	External beam radiation totaling 24 grays was administered over 12 fractions.
							Subperiosteal dissection was performed. The extent of removal and the locations of the inferior surface of the right occipital condyle and the superior surface of the right C2 facet were evaluated by navigation.		
							A mesh titanium interbody cage of the appropriate size was used. Bilateral C2 pedicle screws and C3 lateral mass screws, along with a sub-occipital plate, were fixed, followed by contoured rod placement.		
							The bony surfaces were decorticated, and allograft bone was applied.		
						The CT angiogram confirmed the co-dominant VAs.			
			Sub-acute saddle anesthesia						
			Upper extremity paresthesia’s and some slight left hemibody weakness						
			Tenderness in the posterior scalp and paraspinal musculature						
			The sensation in the left upper and lower extremities was reduced without a dermatomal pattern.						
									Follow-up remained uneventful.
			The slight left Hoffman’s reflex						

Only abstract is available; NA*, not available.

Various treatment options, including resection, selective arterial embolization, percutaneous intralesional injection of calcitonin and methylprednisolone, and subcutaneous injection of denosumab, have been successfully attempted in atlas vertebrae ABCs^[27-29]^. However, they may need multiple therapy sessions that take effect over several months. Selective arterial embolization in the upper cervical region is accompanied by the risk of spinal cord infarction^[11]^. In one case, ossification of the posterior arch of C1 after treatment with calcitonin and methylprednisolone led to occipital neuralgia, which ultimately required surgical resection^[30]^.

### Anatomical considerations in ABC of atlas surgery

Two-fifths of the atlas ring comprises the lateral masses^[31]^. The average midportion dimensions are as follows: height 15.68 mm (SD 0.98), length 16.82 mm (SD 1.0), and width 16.06 mm (SD 0.91). The transverse ligament tubercles located on the medial aspect of the lateral masses play a role as the lateral insertion point for the ligament^[32]^. equal diameter left and right Vas are present in 25%–40.8% of the normal population. Dominance was reported in 35.8%–50% and 23.4%–25% of the left and right VA, respectively^[31,32]^. The left VA is hypoplastic in 5.7% and absent in 1.8%, while the right VA is hypoplastic in 8.8% and absent in 3.1%. The VA anomaly incidence rate falls in a range between 3.5% and 19.2%^[31,32]^.

In the operative corridor for C1 lateral mass reconstruction/lateral mass screw insertion, it was suggested to consider the following anatomical variations^[32,33]^:

- Ponticulus posticus is present in up to 15.6% of cases. It is a bony covering of the V3 segment of the VA located on the posterior arch of C1^[32]^.
- The primitive first cervical intersegmental artery is observed unilaterally in 3.8% and bilaterally in 0.8% of cases^[32]^
- The posterior inferior cerebellar artery (PICA) with an extradural origin is identified in 5%–20% of cases, and it may arise from V3 or even V2^[32]^.
- In 46%, the posterior spinal artery may arise from V3 or from an extradural PICA^[32]^.

### Surgical consideration in ABC of atlas

Surgical management of atlas ABCs is associated with significant challenges due to the unique anatomy and proximity of neurovascular structures in this region, especially the brainstem, spinal cord, and VAs. We encountered two main concerns: preservation versus sacrificing the VA and properly stabilizing and reconstructing the craniocervical junction while achieving the GTR.

A complete preoperative evaluation of the VA using digital subtraction angiography is warranted to determine its course, dominance, and blood flow tolerance via the BOT to preserve the VA. Some surgeons may prefer VA occlusion to achieve GTR on BOT tolerance or in the presence of significant VA narrowing^[34]^.

Recognized indicators of instability are:

- Tumoral involvement or surgical resection of bony structures at the craniovertebral junction can result in instability, with axial rotation exceeding 8 degrees of O-C1 to one side^[35]^.
- Craniovertebral junction stabilization is recommended when >50%^[32,35]^ or 75%^[34]^ of an occipital condyle is resected.
- Instability may arise from C1 lateral mass resection due to transverse ligament incompetency^[36,37]^.
- Resection of C1 lateral mass beyond the “prime meridian” leads to the downward and posterior slope of the occipital condyle over the atlas^[36]^
- Bilateral tumoral involvement or resection of C1 lateral mass results in the complete destruction of the weight transfer pathway^[36]^.

According to the spinal instability neoplastic score^[38]^, an osteolytic lesion of C1 lateral mass accompanied by pain would receive a minimum score of 7 (3 for junctional location, 2 for pain, and 2 for lytic lesion), indicating impending or actual instability requiring surgical intervention. The posterior occipitocervical instrumentation with multiple anchoring choices is conventionally employed to reestablish stability in the craniovertebral junction.

In addition to posterior occipitocervical fixation for unstable pathological conditions, C1 lateral mass reconstruction is suggested in cases involving resection beyond the meridian and bilateral tumoral involvement or C1 lateral masses resection. The chance of neurological deficits following VA sacrifice was reported as 6%^[37]^. VA preservation also minimizes the number of levels involved in fusion. When the ipsilateral VA was preoperatively occluded, contralateral C2 pedicle screw insertion is not recommended as it may raise the potential risk to the remaining VA. Extending occipitocervical fixation to include the lateral masses of C3 and C4 is required^[39]^. C1 lateral mass reconstruction can be performed via a direct lateral, posterior, or modified far lateral approach.

The posterior approach provides a comparatively safe option for the following reasons:

- Spine surgeons are well-versed in this approach.
- VA release can be achieved early.
- Both stabilization and reconstruction are conducted using a single posterior approach.

However, lateral approaches involve 2 stages: one for reconstruction and the other for stabilization^[40,41]^. The accessory nerve and the internal jugular vein are encountered in lateral or modified far lateral approaches before reaching the C1 transverse foramen^[40]^. Consequently, these approaches pose an increased risk of harm to the primary arteries, lower cranial nerves, and potential VA injury. Spacers such as titanium mesh cages, employed in C1 lateral mass reconstruction, enhance fusion rates and reduce the risk of instrumentation failure by distributing the load and alleviating pressure on posterior stabilization devices. The expandable cage provides additional secure embedding through primary and eventual size adjustments.^[37]^.

Intraoperatively, considering GTR as the primary goal, the surgeon should decide whether to preserve or sacrifice the patient’s VA. To safeguard the VA, one should consider skeletonizing and mobilizing before tumor resection^[42]^. Since VA blood flow has tolerance in the BOT and the aim for en bloc resection, we preferred the right VA ligation to achieve GTR. We exposed the proximal part of the V3 segment in the exit point of C2 transverse foramina and ligated the right VA using hemoclips. The second clip was placed at the entry point of the VA to the intradural segment. Due to the ligation of the right VA, no pedicle or lateral mass screw was inserted on the left side to avoid potential injury to the VA. After preserving or sacrificing VA and total tumor resection, the final step is stabilizing and reconstructing the C1 lateral mass. C1 has 2 lateral masses articulating with the superior surface of the articular pillar of the axis and occipital condyle, which transmits the load from the occipital condyle to the C2^[6]^. Hence, lateral mass destruction can result in load-bearing failure and instability^[31,43]^. Accordingly, the most challenging part of the surgery is craniocervical stabilization and reconstruction. In our case, we inserted a bilaterally placed laminar C2 screw to avoid possible left VA artery injury. According to Ma et al.^[44]^ study, it has the same mechanical profile compared to C2 pedicular screw.

Knowledge from the literature addresses craniocervical stabilization using an iliac crest graft, rib graft, or expandable cage (Table 2).

**Table 2:** Previous reports of craniocervical stabilization techniques in ABC surgeries

	Author/year	Age/gender	Region of C1 involvement	Management	Fixation technique	Outcome
1	Canlorbe P^[49]^/1961	NA^*^	NA	NA	NA	NA
2	Pouyanne, H^[50]^/1961	NA	Posterior arch	NA	NA	NA
3	Legre, J^[51]^	NA	Posterior arch	NA	NA	NA
4	Bongioanni F.^[9]^/1996	36/M	Right lateral mass	Tumor resection via anterolateral approach	Iliac crest bone between OC condyle and C2 lateral mas without fixation	Favorable
5	Gladden ML^[27]^/2000	4/F	Right lateral mass	CT-guided aspiration followed by injection of 2 cc methylprednisolone acetate 40 mg and Calcitonin 200 IU	None	An approximately 30% increase in intralesional calcification was found. No change in the lesion’s overall size was noted.
						Treatment was repeated after 6 months of the initial injection
6	Mohit AA^[28]^/2004	10/F	Right lateral mass	Three steps of embolization with 150 to 250 µm of polyvinyl alcohol particles	None	Near complete ossification of C1 after 18 months without recurrence
7	Rai AT^[30]^/2005	11/F	Posterior arch	CT-guided aspiration followed by:	None	After 6 months, cervical laminectomy and tumor resection were done because of occipital neuralgia due to the mass effect of bone ossification
				Intralesional injection of methylprednisolone acetate 125 mg and Calcitonin 200 IU		
				Intralesional injection bone putty		
8	Wang VY/2009^[6]^	12/F	Right lateral mass and anterior arch	Preoperative endovascular ligation of the right VA and GTR of the tumor via the posterior approach	Occiputocecervical fixation and right lateral mass reconstruction with mesh cage fusion with autologous bone and C2 sublaminar wire	Favorable outcomes without recurrence during 6 months follow-up.
9	Patel R, S^[29]^/2018	16/M	Right lateral mass and posterior arch	CT-guided aspiration followed by:	None	Pain-free after 7 months and with no recurrence by 1 year following completion of treatment
				Intralesional injection of methylprednisolone acetate 80 mg and Calcitonin 400 IU		
				120 mg Denosumab was administrated subcutaneously once a month for one year		
10	Mousavi, R^[37]^		Case 1: Right lateral mass extended to the anterior arch with minimal invasion to the spinal	Intra-lesional resection followed by right lateral mass reconstruction using an expandable cage and VA preservation for both cases.	One stage posterior occipitocervical fixation	On postoperative month 3, both patients remained pain-free and sustained favorable control radiographs.
			Canal with no compression effect. Case 2: right lateral mass with extradural extension to the spinal canal			

A recent report described the painful torticollis due to unilateral destruction of the lateral mass secondary to tuberculosis. A tricortical iliac bone graft was used, secured with a C2 pedicle screw-C1 hook on the side of destruction and C1-C2 harms on the contralateral side^[45]^. However, impairment of the C1 lateral mass and posterior arch in our patients led to complete destruction, affecting the C1 anterior/posterior arch and partially the occiput condyles. Hence, C1-C2 stabilization could not be yielded, prompting the use of an appropriately sized titanium mesh cage.

Titanium cages have been widely used to reconstruct the cervical vertebral body after a corpectomy^[46,47]^. Biomechanical investigations have demonstrated that reconstruction using a titanium mesh cage is biomechanically as efficient as other structural grafts like expandable cages^[47]^. In addition, we can administrate a larger volume of the allogeneic graft using an expandable cage with the expectation of more favorable fusion outcomes. Instrumentation and fusion from the occiput to the C2 vertebra offer additional stability.

## Limitations

We acknowledge the low sample size included in this case series. Further reports are highly warranted to corroborate the current findings in a broader population.

## Conclusion

The treatment of choice for C1 ABC is GTR of the lesion and subsequent reconstruction of C1. However, it is very challenging. We successfully performed a GTR of our patients’ ABCs of the C1 lateral mass via a posterior approach and reconstructed the C1 lateral mass using a titanium mesh cage. Our described method also applies to other resection surgeries of the craniocervical junction.

## Data Availability

Not applicable.
